# A nationwide survey of hospital pharmacist interventions to improve polypharmacy for patients with cancer in palliative care in Japan

**DOI:** 10.1186/s40780-019-0143-5

**Published:** 2019-07-03

**Authors:** Mayako Uchida, Shinya Suzuki, Hideki Sugawara, Yukio Suga, Hideya Kokubun, Yoshihiro Uesawa, Takayuki Nakagawa, Hisamitsu Takase

**Affiliations:** 1 Education and Research Center for Clinical Pharmacy, Osaka University of Pharmaceutical Sciences, 4-20-1 Nasahara, Takatsuki, Osaka, 569-1094 Japan; 2 Department of Pharmacy, National Cancer Center Hospital East, 6-5-1, Kashiwanoha, Kashiwa, Chiba, 277-8577 Japan; 3 Department of Pharmacy, Kagoshima University Hospital, 8-35-1 Sakuragaoka, Kagoshima, Kagoshima 890-8520 Japan; 4 Department of Clinical Drug Informatics, Faculty of Pharmacy, Institute of Medical, Pharmaceutical & Health Science, Kanazawa University, 13-1, Takaramachi, Kanazawa, Ishikawa 920-8641 Japan; 5 Tokyo University of Pharmacy and Life Sciences, 1432-1, Horinouchi, Hachioji, Tokyo, 192-0392 Japan; 6 Department of Medical Molecular Informatics, Meiji Pharmaceutical University, 2-522-1 Noshio, Kiyose, Tokyo, 204-8588 Japan; 7 Department of Clinical Pharmacology and Therapeutics, Kyoto University Hospital, 54 Shogoin-Kawahara-cho, Sakyo-ku, Kyoto, 606-8507 Japan; 8 Nippon Medical School Tama-Nagayama Hospital, 1-7-1, Nagayama, Tama-shi, Tokyo, 206-8512 Japan; 9 Research Promotion Committee, Japanese Society for Pharmaceutical Palliative Care and Sciences (JSPPCS), Osaka, Japan

**Keywords:** Polypharmacy, Palliative care, Nationwide survey, Opioids, Intervention, Board-certified pharmacists

## Abstract

**Background:**

There is no nationwide data on polypharmacy in palliative care in Japan. In this study, the research committee of the Japanese Society for Pharmaceutical Palliative Care and Sciences conducted an online survey on polypharmacy and inappropriate prescriptions involving its members who worked as hospital pharmacists.

**Methods:**

The online questionnaire included questions about hospital pharmacist interventions for cancer patients who regularly used six or more drugs during a two-month period from October to November 2017.

**Results:**

Of 2618 hospital pharmacists, 359 responded (13.7%). With regard to cancer patients receiving opioids, 40.9 and 22.3% of the respondents replied that percentages of patients prescribed six or more regular medications were “40–69%” and “70–99%,” respectively. Regarding patients on polypharmacy, 73.0% of the respondents reported a low or moderate rate of inappropriate prescriptions, with responses such as “long-term administration of irresponsible or aimless medications”, “adverse drug reactions,” and “duplication of the pharmacological effect”. Furthermore, 24.2, 46.8, and 23.4% of respondents replied that the rates of drug reduction due to pharmacist recommendations were “0”, “1–39%”, and “more than 40%,” respectively. Pharmacist interventions decreased the use of inappropriate medications, including antiemetics, gastrointestinal medications, and hypnotic sedatives, and reduced or prevented adverse drug reactions such as extrapyramidal symptoms, delirium, and sleepiness. Similar results were obtained for cancer patients who did not use opioids. However, the rates of cancer patients on polypharmacy and with reduction of inappropriate medications by pharmacist interventions were significantly higher in cancer patients receiving opioids. Finally, recommendations of board-certified pharmacists in palliative pharmacy contributed to a decrease in the use of inappropriate medications in cancer patients on polypharmacy (*p* = 0.06).

**Conclusion:**

This nationwide survey clarified pharmacist interventions for polypharmacy in palliative care in Japan. Our data showed frequent polypharmacy in cancer patients receiving opioids, and benefits of pharmacist interventions, especially by board-certified pharmacists in palliative pharmacy, for reducing inappropriate medications and improving adverse drug reactions.

**Trial registration:**

The study approval numbers in the institution; 0046. Registered November 6, 2017.

**Electronic supplementary material:**

The online version of this article (10.1186/s40780-019-0143-5) contains supplementary material, which is available to authorized users.

## Introduction

Polypharmacy was defined 150 years ago, and it has been cited and addressed as an important issue since the 1960s [1]. Polypharmacy is generally defined as above a specific number of regular use medications or as inappropriate or unnecessary uses of multiple medications, such as lack of indication, lack of efficacy, therapeutic duplication, long-term administration of irresponsible or aimless medications, or overdose to the patients [2–4]. Although there is no clear consensus on the number of medications [4], many reports define five or more, or six or more regular use medications as polypharmacy [5–8]. Several studies have reported that the rate of polypharmacy to be approximately 40% (defined as ≥9 medications) from a survey of over 13,000 nursing home residents in the United States [9], 45% (defined as ≥5 medications) from a survey of patients over the age of 75 years in the emergency department in the United Kingdom [10], and 50 to 70% (defined as ≥5 medications) from a survey of inpatients over the age of 65 years in internal medicine wards in Italy [11]. Polypharmacy potentially associated with inappropriate prescriptions, and causes various problems such as drug interactions, adverse events, increased medical expenses, and decreased medication adherence [12] and has been considered a problem in Japan in recent years. An observational survey conducted by a visiting pharmacist revealed the rate of inappropriate prescription was 48% in older patients in Japan [13]. Regular use of six or more medications [14, 15] and five or more medications [16] was associated with the increase in adverse drug reactions and decreased patient adherence [17, 18]. Furthermore, a dose-dependent relationship between polypharmacy and mortality is observed, and excessive polypharmacy (i.e., regular use of ten or more medications) is associated with death [19]. On the other hand, recent evidence suggests that deprescribing, a process of identifying and discontinuing inappropriate medications, can reduce inappropriate polypharmacy in older patients, although it is uncertain whether it can improve clinical outcomes [20, 21].

Hospital pharmacists conduct clinical drug evaluations in inpatients. Interventions in polypharmacy are among the most important tasks and an important obligation required of pharmacists. However, management of polypharmacy still remains a challenge for most hospital pharmacists in Japan. Little evidence exists regarding the effects of a pharmacist interventions on polypharmacy in Japanese clinical practice settings.

As cancer patients inevitably experience many events and need many medications, cancer-related therapies may frequently become polypharmacy [22], and caution against the prescription of multiple drug combinations is required in cancer patients and the elderly [23]. Especially in palliative care for cancer patients, it is quite likely for patients to be on polypharmacy because of the use of a number of drugs for symptomatic relief. In addition, the use of opioids for cancer pain relief and its supportive medications such as gastrointestinal medications and antiemetics may increase polypharmacy [24]. However, even in cancer patients in palliative care, polypharmacy is a high risk for the occurrence of inappropriate prescriptions that should be identified and reduced by pharmacists. Nevertheless, to date, no nationwide data are available on polypharmacy and inappropriate prescriptions in palliative care in Japan. Therefore, the research committee of the Japanese Society for Pharmaceutical Palliative Care and Sciences (JSPPCS) conducted a survey on polypharmacy and inappropriate prescriptions for its members who worked as hospital pharmacists without obtaining patient’s personal information. The purpose of this study was to clarify hospital pharmacist interventions and their effects on polypharmacy in cancer patients who did or did not receive opioids in their routine work in Japan. Especially, we evaluated the benefits of the interventions of a Board-Certified Pharmacist in Palliative Pharmacy (BCPPP), an accreditation offered by the JSPPCS since 2009, on polypharmacy in cancer patients.

## Materials and methods

### Study design and data source

The survey subjects were 2618 hospital pharmacists across Japan who were members of the JSPPCS. We conducted the questionnaire survey between January and February 2018. We asked the pharmacists about polypharmacy and their interventions for patients with cancer between October and November 2017. The research committee sent an e-mail that explained the purpose of the questionnaire study, advertised the survey on the website, and requested all members to take the survey. Respondents answered the survey questions by checking the medical and prescribing records in their hospitals during the investigation period. There were no rewards offered for responses, and thus, taking the survey constituted voluntary work.

### Definitions

#### Definitions of polypharmacy

In this study, we defined polypharmacy based only on the number of medications to analyze the results uniformly collected from various states of hospital pharmacists in their routine work. A systematic review [19] reported that the definition of polypharmacy used in studies can be classified as 1–4, 5, 6–9, or 10 or more medications. Polypharmacy is often defined as the regular use of five or more medications. However, it is increasingly acceptable that multiple medications can be appropriate under certain circumstances [25], such as palliative care. In this study, we defined “polypharmacy” as the regular use of six or more medications (not including p.r.n. medications), as this number of medications is significantly associated with an increase in adverse drug reactions in Japan [16]. It is noted that hospital pharmacists can receive a healthcare reimbursement fee from the national insurance when they reduce two or more drugs for patients prescribed six or more regular medications in the Japanese medical-service fee system “Total drug evaluation and management healthcare reimbursement fee”.

#### Definitions of inappropriate prescribing and medication use

There are several definitions for inappropriate prescribing [26–31]. The American Geriatrics Society Beers Criteria [29] and Screening Tool of Older People’s Prescriptions (STOPP) [28] are well-known criteria that address multiple elements for reducing polypharmacy. However, in this study, we defined “inappropriate prescribing and medication use” as: 1) therapeutic duplication, or the prescription of multiple medications for the same indication or the same class of medications; 2) the prescription of medications that may cause clinically significant drug-drug or drug-disease interactions; 3) wrong dosage, frequency, duration and routes of administration of medications; 4) long-term administration of irresponsible or aimless medications, and; 5) the prescription of medications that may increase the risk of occurrence of adverse drug reactions. The “inappropriate prescribing and medication use” and its causes were judged by the respondents. Inappropriate medications did not include p.r.n. medications.

#### Definitions of regular medication

In this study, we defined “regular medication” as a prescribed medication to take on schedule, except for p.r.n. medication taken only when symptoms occur.

### Questionnaire

Eight members of the research committee of the JSPPCS first developed the draft version of questionnaires and options for answers. Before starting the nationwide survey, we conducted a pilot investigation for the members and 13 co-workers with over 10 years of clinical pharmacist experience to validate the draft questionnaire. Based on the results and suggestions/comments from the pilot investigation, we modified and adjusted the questionnaires and options for answers that were finalized under the agreement and understanding of all members of the research committee (13 members) and executive board of the JSPPCS (20 members).

The questionnaire (Additional file 1: Table S1) was administered using the society’s website (URL: http://jpps.umin.jp/kenkyu/index.html). In the first section, we investigated: 1) sex of the respondents (options); 2) years of pharmacist experience (options); 3) receipt or not of the “Total drug evaluation and management healthcare reimbursement fee” in the institution (options); 4) pharmacy board certification (options, multiple answers allowed); 5) confidence in palliative care (options); 6) number of continuing education sessions related to palliative care attended in the last year (options), and; 7) the percentage of cancer patients in all the patients managed by the pharmacist (options). The confidence score ranged from zero (no confidence) to ten (full confidence), and was self-evaluated by the respondent, as previously described [32]. As part of the questionnaire survey, the respondents were also asked about their board certifications related to cancer therapy and palliative care, such as the JSPPCS certification BCPPP, the Japanese Society of Pharmaceutical Health Care and Sciences (JSPHCS) certification of Oncology Pharmacist, the Japanese Society of Hospital Pharmacists certification of Board Certified Pharmacist in Oncology Pharmacy (BCPOP), the Japanese Society of Pharmaceutical Oncology certification of Accredited Pharmacist of Ambulatory Cancer Chemotherapy (APACC), and other board pharmacy certifications available in Japan.

In the second and third sections, we investigated the pharmacist interventions in polypharmacy for cancer patients who did and did not receive opioids, respectively, as follows: 1) number of opioid-using cancer patients managed by the respondents in the two-month study period; 2) percentage of opioid-using cancer patients who were prescribed six or more regular medications (options); 3) percentage of inappropriate prescriptions in the cases of the patients on polypharmacy (options); 4) reasons for inappropriate prescriptions identified by pharmacists (options, multiple answers allowed); 5) percentage of patients on polypharmacy with drug reduction due to a pharmacist recommendation (options); 6) reasons for inappropriate prescription reduction based on the pharmacist recommendation (options, multiple answers allowed); 7) number of concurrent regular medications reduced due to a pharmacist recommendations (options); 8) drugs reduced due to a pharmacist recommendations (options, multiple answers allowed), and; 9) reduced symptoms of adverse drug reactions because of pharmacist recommendations (free description).

### Exclusion criteria

When the respondents did not answer some questions, we excluded only the blank data, but included other available data from questions that were answered by the respondents.

### Data analysis

When compared between the opioid-using and non-using patients, bivariate analyses were employed to examine differences in the demographic characteristics, using chi-square tests for categorical variables. Nonparametric multiple comparison analyses followed by the Steel-Dwass’s test were performed to examine the correlation between board certifications of pharmacists in cancer therapy and palliative care. All data were analyzed using JMP Pro version 13.2.0 (SAS Institute, Cary, NC, United States). A *p*-value < 0.05 and a *p*-value < 0.10 were considered as statistically and marginally significant, respectively.

## Results

### Response rates and subjects’ backgrounds

Of 2618 hospital pharmacists, 359 responded to the survey, and the response rate was 13.7%. As shown in Table 1, the percentage of respondents who had more than 10 years of experience as a pharmacist was 73.5% (264/359). Forty-nine percent of respondents replied that their facilities had received the “Total drug evaluation and management healthcare reimbursement fee”. Of the 359 respondents, 222 (61.8%) had some board pharmacy certification related to cancer therapy and palliative care and 130 (36.2%) had no board certification. The top four board certifications were as follows: BCPPP offered by the JSPPCS (*n* = 123, 34.3%), BCPOP (*n* = 82, 22.8%), the JSPHCS certification of Oncology Pharmacist (*n* = 52, 14.5%), and APACC (*n* = 32, 8.9%). The total number of other board-certified pharmacists with certifications other than BCPPP was 99 (27.6%). The score of confidence in palliative care was widely distributed on a scale of zero to 10, and the median score was seven. More than 95% of the respondents had attended at least one continuing education event related to palliative medication in the past one year. Many patients managed by the respondents were patients with cancer: 44.9% of respondents replied that more than 70% of patients were cancer patients.

**Table 1:** Background characteristics of respondents

		*n*	(%)
Sex	Male	200	(55.7)
	Female	158	(44.0)
	No response	1	(0.3)
Pharmacy experience, years	1 to 3	8	(2.2)
	4 to 6	42	(11.7)
	7 to 9	44	(12.3)
	10 to 14	94	(26.2)
	15 to 19	65	(18.1)
	more than 19	105	(29.2)
	No response	1	(0.3)
Receives “Total drug evaluation and management healthcare reimbursement fee”			
	Yes	176	(49.0)
	No	183	(51.0)
Board pharmacy certification	Yes	222	(61.8)
	No	130	(36.2)
	No response	7	(1.9)
	BCPPP^1)^	123	(34.3)
	BCPOP^2)^	82	(22.8)
	JSPHCS^3)^ certification of Oncology Pharmacist	52	(14.5)
	APACC^4)^	32	(8.9)
	Pharmacists with certifications other than BCPPP	99	(27.6)
Confidence score in palliative care (No confidence, 0; full confidence, 10)			
	Zero	5	(1.4)
	1	7	(1.9)
	2	5	(1.4)
	3	9	(2.5)
	4	10	(2.8)
	5	54	(15.0)
	6	76	(21.2)
	7	92	(25.6)
	8	69	(19.2)
	9	24	(6.7)
	10	8	(2.2)
Nationwide attendance at continuing education sessions related to palliative care in a year			
	Zero	17	(4.7)
	1 to 3	260	(72.4)
	4 to 6	58	(16.2)
	7 to 9	14	(3.9)
	More than 9	10	(2.8)
Percentage of cancer patients managed by pharmacists			
	Zero	7	(1.9)
	1 to 39%	97	(27.0)
	40 to 69%	94	(26.2)
	70 to 99%	103	(28.7)
	100%	58	(16.2)

1) BCPPP: Board Certified Pharmacist in Palliative Pharmacy

2) BCPOP: Board Certified Pharmacist in Oncology Pharmacy

3) JSPHCS: Japanese Society of Pharmaceutical Health Care and Sciences

4) APACC: Accredited Pharmacist of Ambulatory Cancer Chemotherapy

### Pharmacist interventions in polypharmacy for cancer patients who used opioids

In the first section, the questionnaire asked respondents about interventions for opioid-using cancer patients (Tables 2 and 3). The median number of opioid-using cancer patients managed by the respondents was 10 (range 1–300). The percentages of opioid-using cancer patients who were prescribed six or more regular medications were as follows: “zero” (7.2%), “1–39%” (21.4%), “40–69%” (40.9%), “70–99%” (22.3%), and “100%” (6.1%). In the cases of the patients on polypharmacy, the percentages of inappropriate prescriptions detected by pharmacists were as follows: “zero” (24.5%), “1–39%” (64.3%), “40–69%” (8.1%), and “70–99%” (0.6%). The top three reasons for inappropriate prescriptions identified by pharmacists were “long-term administration of irresponsible or aimless medications” (63.8%), “adverse drug reactions caused by medications” (24.0%), and “medications-mediated duplication of the pharmacological effect” (21.7%). The percentages of patients on polypharmacy with drug reduction due to a pharmacist recommendations were “none” (24.2%), “1–39%” (46.8%), “40–69%” (12.0%), “70–99%” (5.0%), and “100%” (6.4%). Thus, 70.2% of respondents reduced the number of inappropriately prescribed drugs in opioid-using cancer patients on polypharmacy. The reasons for inappropriate prescriptions reduced by pharmacist recommendations were “long-term administration of irresponsible or aimless medications” (58.8%), “adverse drug reactions caused by medications” (38.4%), “change from oral to other dosage form due to oral feeding difficulty” (35.1%), “medication-mediated duplication of the pharmacological effect” (24.8%), “medication-induced drug-drug interactions” (15.9%), and “other” (5.0%).

**Table 2:** Pharmacist interventions for cancer patients who used opioids

		*n*	(%)
Number of opioid-using cancer patients			
	Median	10	
	[Range]	[1–300]	
Percentage of opioid-using cancer patients prescribed six or more regular medications			
	Zero	26	(7.2)
	1 to 39%	77	(21.4)
	40 to 69%	147	(40.9)
	70 to 99%	80	(22.3)
	100%	22	(6.1)
	No response	7	(1.9)
Percentage of inappropriate prescriptions in opioid-using cancer patients prescribed six or more regular medications			
	Zero	88	(24.5)
	1 to 39%	231	(64.3)
	40 to 69%	29	(8.1)
	70 to 99%	2	(0.6)
	100%	0	(0)
Reasons for inappropriate prescriptions (multiple answers from options)			
	Long-term administration of irresponsible or aimless medications	229	(63.8)
	Adverse drug reactions caused by medications	86	(24.0)
	Medications-mediated duplication of the pharmacological effect	78	(21.7)
	Medication-induced drug-drug interactions	46	(12.8)
	Other	24	(6.7)
Percentage of patients on polypharmacy with drug reduction due to pharmacist recommendations			
	Zero	87	(24.2)
	1 to 39%	168	(46.8)
	40 to 69%	43	(12.0)
	70 to 99%	18	(5.0)
	100%	23	(6.4)
	No response	20	(5.6)
Reasons for pharmacist recommendations to reduce medications (multiple answers from options)			
	Long-term administration of irresponsible or aimless medications	211	(58.8)
	Adverse drug reactions caused by medications	138	(38.4)
	Change from oral to other dosage form due to oral feeding difficulty	126	(35.1)
	Medications-mediated duplication of the pharmacological effect	89	(24.8)
	Medication-induced drug-drug interactions	57	(15.9)
	Other	18	(5.0)
Average number of medications reduced by pharmacist recommendations			
	0	55	(15.3)
	1	154	(42.9)
	2	81	(22.6)
	3	18	(5.0)
	4	1	(0.3)
	More than 4	4	(1.1)

**Table 3:** Reduced drugs and improved adverse drug reactions due to pharmacist interventions for cancer patients who used opioids

		*n*	(%)
Pharmacological categories of drugs reduced by pharmacist recommendations			
	Antiemetics	161	(44.8)
	Gastrointestinal medications	141	(39.3)
	Hypnotic sedatives	103	(28.7)
	Analgesics	102	(28.4)
	Laxatives	72	(20.1)
	Antipsychotics	51	(14.2)
	Other	43	(12.0)
Reduced drugs in each pharmacological category (multiple answers from options)			
Antiemetics	Dopamine receptor antagonists	119	(33.1)
	Prokinetic agents	79	(22.0)
	Antihistaminic agents	20	(5.6)
	Other	2	(0.6)
Gastrointestinal medications	Histamine H_2_ receptor blockers	69	(19.2)
	Proton pump inhibitors	48	(13.4)
	Gastric antacids	37	(10.3)
	Prostaglandin analogs	16	(4.5)
	Other	30	(8.4)
Hypnotic sedatives	Benzodiazepines	103	(28.7)
	Non-benzodiazepines	31	(8.6)
Analgesics	Non-steroidal anti-inflammatory drugs	60	(16.7)
	Analgesic adjuvants	40	(11.1)
	Opioids	32	(8.9)
	Acetaminophen	22	(6.1)
Laxatives	Salt-based laxative	49	(13.6)
	Peroral stimulative laxatives	25	(7.0)
	Enema clysters	6	(1.7)
	Small intestine irritant laxative	3	(0.8)
	Other	3	(0.8)
Antipsychotics	Typical antipsychotics	51	(14.2)
	Atypical antipsychotics	32	(8.9)
Others	Hypertensives	14	(3.9)
	Vitamins	8	(2.2)
	Antidiabetics	5	(1.4)
	Chinese herbal medicine	4	(1.1)
	Medication for intestinal disorders	4	(1.1)
	Drugs to facilitate urination via the bladder	4	(1.1)
	Drugs for high cholesterol	4	(1.1)
	Cardiovascular drugs	2	(0.6)
	Cold medicines	2	(0.6)
	Anticoagulants	2	(0.6)
	Steroids	2	(0.6)
	External medicine	2	(0.6)
	Antihyperuricemics	2	(0.6)
	Diuretics	1	(0.3)
	Antibiotics	1	(0.3)
	Antiasthmatic drugs	1	(0.3)
	Antiepileptic drugs	1	(0.3)
	Antidementia drugs	1	(0.3)
	Antivirals	1	(0.3)
	Anticancer drugs	1	(0.3)
	Infusion fluid	1	(0.3)
	Other	2	(0.6)
Symptoms improved due to pharmacist interventions (free multiple answers)			
	Extrapyramidal symptoms	100	(27.9)
	Delirium	49	(13.6)
	Sleepiness	36	(10.0)
	Constipation	20	(5.6)
	Renal dysfunction	18	(5.0)
	Dizziness	16	(4.5)
	Nausea and vomiting	14	(3.9)
	Electrolyte abnormalities	11	(3.1)
	Sleep disorders	7	(1.9)
	Gastrointestinal disorders	7	(1.9)
	Hypotension	6	(1.7)
	Liver dysfunction	4	(1.1)
	Hypoglycemia	3	(0.8)
	Bleeding	3	(0.8)
	Myasthenia gravis	2	(0.6)
	Edema	2	(0.6)
	Tiredness	1	(0.3)
	Dehydration	1	(0.3)
	Disturbance of consciousness	1	(0.3)
	Dysuria	1	(0.3)
	Leukopenia	1	(0.3)
	Aspiration	1	(0.3)
	Sedation	1	(0.3)
	Stomatitis	1	(0.3)
	Hyperglycemia	1	(0.3)
	Respiratory depression	2	(0.6)

The average numbers of concurrent regular medications reduced due to pharmacist recommendations were “zero” (15.3%), “one” (42.9%), “two” (22.6%), “three” (5.0%), “four” (0.3%), and “five or more” (1.1%). The top three pharmacological categories of the drugs reduced due to pharmacist recommendations were “antiemetics” (44.8%), “gastrointestinal medications” (39.3%), and “hypnotic sedatives” (28.7%). The majority of the drugs reduced in each pharmacological category were as follows: “dopamine receptor antagonists” (33.1%) and “prokinetic agents” (22.0%) among antiemetics, “histamine H_2_ receptor blockers (H_2_ blockers)” (19.2%) among gastrointestinal medications, “benzodiazepines” (28.7%) among hypnotic sedatives, “nonsteroidal anti-inflammatory drugs (NSAIDs)” (16.7%) among analgesics, “salt-based laxative” (13.6%) among laxatives, and “typical antipsychotics” (14.2%) among antipsychotics. The top three symptoms of adverse drug reactions reduced because of pharmacist recommendations were “extrapyramidal symptoms” (27.9%), “delirium” (13.6%), and “sleepiness” (10.0%).

### Pharmacist interventions in polypharmacy for cancer patients who did not use opioids

In the second section, the questionnaire asked about the respondents’ interventions for patients who did not receive opioids (Tables 4 and 5). The median number of opioid non-using cancer patients managed by the respondents was 20 (range, 1–300). The percentages of opioid non-using cancer patients who were prescribed six or more regular medications were as follows: “zero” (10.3%), “1–39%” (39.0%), “40–69%” (34.8%), “70–99%” (8.1%), and “100%” (2.2%). Among the patients on polypharmacy, the percentages of inappropriate prescriptions detected by pharmacists were as follows: “zero” (25.6%), “1–39%” (59.3%), “40–69%” (7.2%), and “70–99%” (0.6%). The top three reasons for inappropriate prescriptions identified by pharmacists were “long-term administration of irresponsible or aimless medications” (56.3%), “medications-mediated duplication of the pharmacological effect” (29.5%), and “adverse drug reactions caused by medications” (20.9%). Percentages of polypharmacy patients with drug reduction due to pharmacist recommendations were “none” (25.9%), “1–39%” (45.4%), “40–69%” (4.2%), “70–99%” (5.8%) and “100%” was (3.3%). Thus, 58.7% of respondents reduced the number of inappropriately prescribed drugs in opioid non-using cancer patients on polypharmacy. The reasons for inappropriate prescriptions reduced by a pharmacist recommendation were “long-term administration of irresponsible or aimless medications” (48.2%), “adverse drug reactions caused by medications” (29.5%), and “medications-mediated duplication of the pharmacological effect” (28.1%), “change from oral to other dosage form due to oral feeding difficulty” (20.6%), “medication-induced drug-drug interactions” (14.5%), and “other” (3.1%).

**Table 4:** Pharmacist interventions for cancer patients who did not use opioids

		*n*	(%)
Number of opioid non-using cancer patients			
	Median	20	
	[Range]	[1–300]	
Percentage of opioid non-using cancer patients prescribed with six or more regular medications			
	Zero	37	(10.3)
	1 to 39%	140	(39.0)
	40 to 69%	125	(34.8)
	70 to 99%	29	(8.1)
	100%	8	(2.2)
	No response	20	(5.6)
Percentage of inappropriate prescriptions in opioid non-using cancer patients prescribed six or more regular medications			
	Zero	92	(25.6)
	1 to 39%	213	(59.3)
	40 to 69%	26	(7.2)
	70 to 99%	2	(0.6)
	No response	26	(7.2)
Reasons for inappropriate prescriptions (multiple answers from options)			
	Long-term administration of irresponsible or aimless medications	202	(56.3)
	Medications-mediated duplication of the pharmacological effect	106	(29.5)
	Adverse drug reactions caused by medications	75	(20.9)
	Medication-induced drug-drug interactions	50	(13.9)
	Other	13	(3.6)
Percentage of patients on polypharmacy with drug reduction due to pharmacist recommendations			
	Zero	93	(25.9)
	1 to 39%	163	(45.4)
	40 to 69%	15	(4.2)
	70 to 99%	21	(5.8)
	100%	12	(3.3)
	No response	55	(15.3)
Reasons for pharmacist recommendations to reduce medications (multiple answers from options)			
	Long-term administration of irresponsible or aimless medications	173	(48.2)
	Adverse drug reactions caused by medications	106	(29.5)
	Medications-mediated duplication of the pharmacological effect	101	(28.1)
	Change from oral to other dosage form due to oral feeding difficulty	74	(20.6)
	Medication-induced drug-drug interactions	52	(14.5)
	Other	11	(3.1)
Average number of medications reduced by pharmacist recommendations			
	0	57	(15.9)
	1	151	(42.1)
	2	53	(14.8)
	3	12	(3.3)
	4	2	(0.6)
	more than 4	5	(1.4)

**Table 5:** Drugs reduced and improved adverse drug reactions due to pharmacist interventions for cancer patients who did not use opioids

		*n*	(%)
Pharmacological categories of drugs reduced by pharmacist recommendations			
	Gastrointestinal medications	126	(35.1)
	Antiemetics	88	(24.5)
	Hypnotic sedatives	83	(23.1)
	Analgesics	81	(22.6)
	Laxatives	60	(16.7)
	Antipsychotics	31	(8.6)
	Other	41	(11.4)
Drugs reduced in each pharmacological category (multiple answers from options)			
Antiemetics	Prokinetic agents	52	(14.5)
	Dopamine receptor antagonists	50	(13.9)
	Antihistaminic agents	15	(4.2)
	Other	5	(1.4)
Gastrointestinal medications	Histamine H_2_ receptor blockers	69	(19.2)
	Proton pump inhibitors	58	(16.2)
	Gastric antacids	33	(9.2)
	Prostaglandin analogs	10	(2.8)
	Other	26	(7.2)
Hypnotic sedatives	Benzodiazepines	81	(22.6)
	Non-benzodiazepines	26	(7.2)
Analgesics	Non-steroidal anti-inflammatory drugs	61	(17.0)
	Analgesic adjuvants	23	(6.4)
	Acetaminophen	21	(5.8)
Laxatives	Salt-based laxative	47	(13.1)
	Peroral stimulative laxatives	21	(5.8)
	Small intestine irritant laxative	3	(0.8)
	Other	2	(0.6)
Antipsychotics	Typical antipsychotics	31	(8.6)
	Atypical antipsychotics	22	(6.1)
Others	Hypertensives	15	(4.2)
	Vitamins	8	(2.2)
	Antibiotics	5	(1.4)
	Drugs for high cholesterol	5	(1.4)
	Medications for intestinal disorders	4	(1.1)
	Diuretics	3	(0.8)
	Antihyperuricemics	3	(0.8)
	Antidiabetics	3	(0.8)
	Chinese herbal medicine	3	(0.8)
	Anticoagulants	1	(0.3)
	Antiepileptic drugs	1	(0.3)
	Gargle medicines	1	(0.3)
	Other	1	(0.3)
Symptoms improved due to pharmacist interventions (free multiple answers)			
	Electrolyte abnormality	20	(5.6)
	Delirium	14	(3.9)
	Hypotension	14	(3.9)
	Renal dysfunction	12	(3.3)
	Extrapyramidal symptoms	9	(2.5)
	Sleepiness	9	(2.5)
	Liver dysfunction	9	(2.5)
	Constipation	8	(2.2)
	Dizziness	8	(2.2)
	Hypoglycemia	4	(1.1)
	Nausea and vomiting	3	(0.8)
	Sleep disorder	3	(0.8)
	Gastrointestinal disorder	3	(0.8)
	Bleeding	2	(0.6)
	Dysuria	2	(0.6)
	Myasthenia gravis	1	(0.3)
	Edema	1	(0.3)
	Tiredness	1	(0.3)
	Dehydration	1	(0.3)
	Hyperglycemia	1	(0.3)
	Fever	1	(0.3)
	Bradycardia	1	(0.3)
	Thrombosis	1	(0.3)
	Digoxin intoxication	1	(0.3)

The average numbers of concurrent regular medications reduced due to a pharmacist recommendations were “zero” (15.9%), “one” (42.1%), “two” (14.8%), “three” (3.3%), “four” (0.6%), and “five or more” (1.4%). The top three pharmacological categories of the drugs reduced due to a pharmacist recommendations were “gastrointestinal medications” (35.1%), “antiemetics” (24.5%), and “hypnotic sedatives” (23.1%). The majority of the drugs reduced in each pharmacological category were as follows: “prokinetic agents” (14.5%) and “dopamine receptor antagonists” (13.9%) among antiemetics, “H_2_ blockers” (19.2%) and “proton pump inhibitors (PPIs)” (16.2%) among gastrointestinal medications, “benzodiazepines” (22.6%) among hypnotic sedatives, “NSAIDs” (17.0%) among analgesics, “salt-based laxative” (13.1%) among laxatives, and “typical antipsychotics” (8.6%) among antipsychotics. The top three symptoms of adverse drug reactions reduced because of a pharmacist recommendations were “electrolyte abnormality” (5.6%), “delirium” (3.9%), and “hypotension” (3.9%).

### Differences in pharmacist interventions in polypharmacy between cancer patients using and not using opioids

The rate of cancer patients who were prescribed six or more regular medications was significantly higher in opioid-using patients than that in opioid non-using patients (*p* < 0.001). However, the rate of inappropriate prescriptions was not statistically different between opioid-using and non-using patients (*p* = 0.906). The top three reasons of inappropriate prescriptions (long-term administration of irresponsible or aimless medications, adverse drug reactions caused by medications, and medications-mediated duplication of the pharmacological effect) were the same between the two groups. The rate of cancer patients on polypharmacy with drug reduction due to pharmacist recommendations was significantly higher in opioid-using patients than that in opioid non-using patients (*p* < 0.01), although the categories of medications reduced by pharmacist recommendations (antiemetics, gastrointestinal medications, and hypnotic sedatives) were the same between the groups. The top three symptoms for adverse drug reactions reduced due to pharmacist recommendations were different: “extrapyramidal symptoms,” “delirium,” and “sleepiness” in opioid-using cancer patients, and “electrolyte abnormality,” “delirium,” and “hypotension” in opioid non-using patients. However, the reduced number of concurrent regular medications was not different between the two groups (*p* = 0.332).

### Correlation between board-certified pharmacists and pharmacist interventions in polypharmacy

**Fig. 1:**
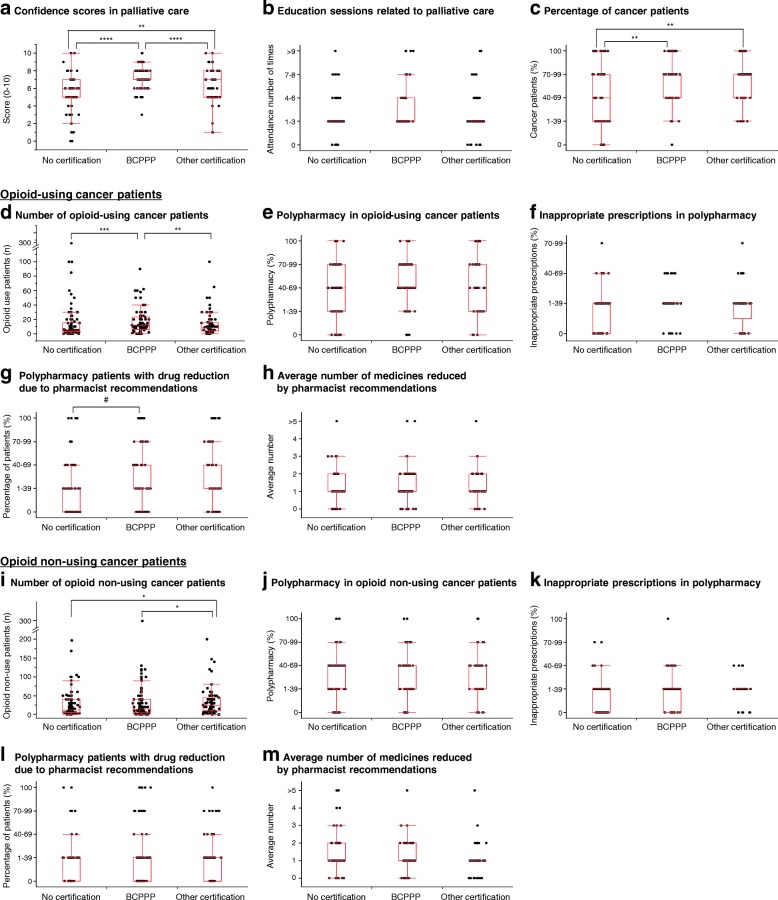
Correlation between the involvement of board-certified pharmacists and pharmacist interventions for polypharmacy. The respondents were divided into three groups; board-certified pharmacist in palliative pharmacy (BCPPP; *n* = 123), other certification (except for BCPPP; *n* = 99), and no-certification groups (*n* = 130). (**a**) Confidence in palliative care (0; no confidence; 10, full confidence); (**b**) attendance at nationwide continuing education sessions related to palliative care in a year; (**c**) percentage of patients with cancer managed by pharmacists (zero, 1–39%, 40–69%, 70–99, and 100%); cancer patients prescribed with opioids (**d**-**h**) and cancer patients prescribed without opioids (I-M) managed by respondents for the two-month study period; (**d** and **i**) number of the patients; (**e** and **j**) percentage of patients prescribed six or more regular medications (zero, 1–39%, 40–69%, 70–99, and 100%); (**f** and **k**) percentage of inappropriate prescriptions in patients on polypharmacy (zero, 1–39%, 40–69%, 70–99, and 100%), (**g** and **l**) percentage of patients on polypharmacy with drug reduction due to pharmacist recommendations (zero, 1–39%, 40–69%, 70–99, and 100%); and (**h** and **m**) average number of medications reduced due to pharmacist recommendations among patients on polypharmacy. Data are expressed as dot-box plot (median, interquartile range, and outliers). *p < 0.05, **p < 0.01, ***p < 0.001, ****p < 0.0001, and #*p* < 0.10 (Steel-Dwass’s test)

We analyzed the correlation between board-certified pharmacists related to cancer therapy and palliative care and pharmacist interventions in polypharmacy (Fig. 1). The respondents were divided into three groups; BCPPP (*n* = 123), other certification (other than BCPPP; *n* = 99), and no-certification groups (*n* = 130). Confidence scores in palliative care in the BCPPP and other certification groups were significantly higher than that in the no-certification group (*p* < 0.0001). Furthermore, the confidence score in the BCPPP group was significantly higher than that in the other certification group (*p* = 0.002) (Fig. 1a). The numbers of attendances at nationwide continuing education sessions related to palliative care in a year were not different among the three groups (Fig. 1b). The percentage of cancer patients managed by the BCPPP and other certification groups were significantly higher than that managed by the no-certification group (*p* < 0.0001) (Fig. 1c).

The number of opioid-using cancer patients managed by the BCPPP group was significantly higher than those managed by the no-certification and other certification groups (*p* = 0.001 and *p* = 0.004, respectively) (Fig. 1d). With regard to opioid-using cancer patients, there were no differences in the rates of polypharmacy and inappropriate prescriptions among the three pharmacist groups (Fig. 1e, f). However, the percentage of polypharmacy cancer patients with drug reduction due to the recommendations by the BCPPP group was marginally higher than that by the no-certification group (*p* = 0.06) (Fig. 1g), although there were no significant differences in the average number of medications reduced by pharmacist recommendations among the three groups (Fig. 1h).

The number of opioid non-using cancer patients managed by the other certification group was significantly higher than those managed by the no-certification and BCPPP groups (*p* = 0.012 and *p* = 0.045, respectively) (Fig. 1i). However, intergroup differences in polypharmacy, inappropriate prescriptions, percentages of polypharmacy cancer patients with drug reduction due to a pharmacist recommendations, and the average number of medications reduced by pharmacists were not significant (Fig. 1j-m).

## Discussion

This is the first nationwide questionnaire survey-based study in Japan showing pharmacist interventions for cancer patients on polypharmacy and inappropriate prescriptions. Remarkably, most of the respondents observed polypharmacy in cancer patients in their charge and 70.2 and 58.7% of respondents have reduced inappropriate medications in opioid-using and non-using cancer patients, respectively, in their routine work.

In this study, we showed that the rate of cancer patients on polypharmacy was higher in opioid-using patients. Polypharmacy is risky even in patients with cancer and older people [23]. Furthermore, cancer patients using opioids tend to be prescribed more medications than opioid non-using patients. This is not surprising since the prescription of opioids is itself an increase in one concurrent medication, and most of opioid-using cancer patients are prescribed non-opioid analgesics, such as acetaminophen and NSAIDs, for the treatment of cancer pain. In addition, supportive medicines such as gastrointestinal medications and laxatives are prescribed for prevention of NSAID-induced gastrointestinal injury and opioid-induced constipation. Thus, the present findings suggest that the use of opioids can further increase the risk for polypharmacy in cancer patients. In cancer patients on polypharmacy, approximately 70% respondents observed some inappropriate prescriptions, such as long-term administration of irresponsible or aimless medications, adverse drug reactions and duplicated pharmacological medications. It is reported that polypharmacy is potentially associated with inappropriate prescriptions [16, 33, 34]. Frequent polypharmacy in cancer patients may cause inappropriate prescriptions. However, in this study, we could not detect a difference in the rate of inappropriate prescriptions between opioid-using and non-using patients, suggesting that it can occur regardless of whether opioids are used or not in cancer patients on polypharmacy.

Approximately 60–70% of respondents contributed to reduce inappropriate medications by pharmacist recommendations in cancer patients on polypharmacy. Thus, hospital pharmacists can actively identify and reduce inappropriate medications, such as long-term administration of irresponsible or aimless medications, adverse drug reactions and duplicated pharmacological medications, in cancer patients on polypharmacy. These pharmacist interventions for polypharmacy could result in resolving or preventing adverse drug reactions related with polypharmacy in cancer patients. In addition, the present results suggest that drug reduction due to pharmacist recommendations was frequent in opioid-using cancer patients rather than opioid non-using cancer patients. It might be due to that larger number of concurrent medications are prescribed, accompanied with the use of opioids, as described above.

In this study, we did not investigate what kinds of medications were frequently and commonly prescribed in cancer patients who did or did not receive opioids. However, the symptoms for adverse drug reactions reduced due to pharmacist recommendations were different between opioid-using and non-using patients. It is reported that antiemetics, gastrointestinal medications, or hypnotic sedatives used concurrently with opioids caused extrapyramidal symptoms or delirium and worsened adverse drug reactions in opioid-using patients [35]. In particular, the contributions were more evident in opioid-using cancer patients for severe adverse drug reactions, such as extrapyramidal symptoms and delirium. The main reason for pharmacist interventions in antiemetic reduction was likely to improve extrapyramidal symptoms. Another reason for pharmacist intervention may be to avoid duplicate prescriptions of H_2_ blockers and PPIs, since H_2_ blockers are known to affect the central nervous system, resulting in delirium in older people [36]. Furthermore, it seemed that pharmacists reviewed the use of antipsychotics or hypnotic sedatives to manage delirium and sleepiness.

The pharmacy certification may be beneficial in managing the appropriate treatment. Board certification was introduced about 10 years ago in Japan following the Western board certification systems. In the United States, the Board of Pharmacy Specialties was established as an organization independent of the American Pharmacists Association. Those board certifications are recognized as surrogate markers for advanced medical practice, such as increased medical knowledge, superior training, and certain aspects of patient care, in general [37]. A previous survey revealed the benefit of board certification in oncology pharmacy in Japan [32]. In this study, we evaluated factors correlated with the BCPPP and other board certifications and showed that board certification had positive effects on the management of polypharmacy. Respondents who possessed the BCPPP and other board certifications had more confidence in palliative care, indicating that certified pharmacists are well experienced in palliative care, although the attendances at education events were similar. Expectedly, the number of cancer patients managed by the BCPPP and other certification groups were higher than those managed by the no-certification group. Interestingly, the number of opioid-using cancer patients managed by the BCPPP group was higher than those managed by the other certification and no-certification groups, suggesting that qualifications, especially BCPPP, motivate hospital pharmacists to use and manage palliative medicines including opioids. Furthermore, the results indicated that BCPPP contributed to a reduction in inappropriate medications among opioid-using patients on polypharmacy. These results suggest the benefits of certification in palliative pharmacy on the improvement of polypharmacy in opioid-using cancer patients.

This study has several limitations. 1) This study is a retrospective survey study investigating the past medication/prescribing records by the respondents. Thus, there was a time lag between the response period of the questionnaire and the investigation period (several months), which may lead to recall bias. However, the questionnaire method was made to broadly clarify the practical situation of hospital pharmacist interventions to improve polypharmacy in their routine work. 2) The response rate to this survey was low (13.7%), although the information was advertised to all members of the JSPPCS via e-mail and through the website. It is possible that the respondents may be a conscientious population motivated to improve polypharmacy, but not representatives of hospital pharmacists in Japan. Therefore, we could not expand the present data to general information for Japanese pharmacists. 3) In the present Japanese medical service fee system, hospital pharmacists can receive a healthcare reimbursement fee from national insurance when they reduce two or more drugs for patients prescribed six or more regular medications. The fee system can motivate pharmacists to reduce inappropriate medications, while it also causes bias to reduce medications in the present retrospective study. 4) The correlation between medical benefit and the improvement of polypharmacy by certified pharmacists remains unclear, as described previously [38, 39]. In this study, we evaluated the clinical pharmacy service only during a two-month period. However, we believe that board certification not only facilitates an appropriate involvement of qualified pharmacists but also contributes toward motivating staff members to improve polypharmacy-related problems. To exclude the biases from the present retrospective analysis, we are planning to conduct a multi-center prospective observational study.

## Conclusions

In this study, we first clarified pharmacist interventions in polypharmacy and inappropriate prescriptions based on a nationwide questionnaire survey in Japan. The findings suggest frequent polypharmacy in cancer patients receiving opioids, and the benefits of pharmacist interventions on not only the reduction of inappropriate medications but also improvement of adverse drug reactions in cancer patients on polypharmacy. Furthermore, pharmacy certifications might be beneficial in improving polypharmacy.

## Additional file

Additional file 1: Questionnaire. (PDF 182 kb)

## Data Availability

The dataset supporting the conclusions of this article is included within the article.

## Abbreviations

APACC: Accredited Pharmacist of Ambulatory Cancer Chemotherapy
BCPOP: Board Certified Pharmacist in Oncology Pharmacy
BCPPP: Board Certified Pharmacist in Palliative Pharmacy
H_2_ blockers: Histamine H_2_ receptor blockers
JSPHCS: Japanese Society of Pharmaceutical Health Care and Sciences
JSPPCS: Japanese Society for Pharmaceutical Palliative Care and Sciences
NSAIDs: Nonsteroidal anti-inflammatory drugs
PPIs: Proton pump inhibitors
