# Supplementary material for: A nationwide survey of hospital pharmacist interventions to improve polypharmacy for patients with cancer in palliative care in Japan
Source: J Pharm Health Care Sci. 2019 Jul 3;5:14. doi: 10.1186/s40780-019-0143-5 (PMC6607579; doi:10.1186/s40780-019-0143-5)
Supplement: Supplementary file 1 — Questionnaire. (PDF 182 kb) [file 40780_2019_143_MOESM1_ESM.pdf]

Additional file 1

Please answer the following questions about yourself

Q1) Sex
1. Male **2.** Female

Q2) Pharmacy experience
1. 1 to 3 years **2.** 4 to 6 years **3.** 7 to 9 years **4.** 10 to 14 years
5. 15 to 19 years **6.** More than 19 years

Q3) "Total drug evaluation and management healthcare reimbursement fee" in the institution
1. Receipt **2.** Not receipt

Q4) Pharmacy board certification (multiple answers allowed)
1. Japanese Society for Pharmaceutical Palliative Care and Sciences certification
2. Japanese Society of Pharmaceutical Health Care and Sciences certification of Oncology Pharmacist
3. Japanese Society of Hospital Pharmacist certification of Board Certified Pharmacist in Oncology Pharmacy
4. Japanese Society of Pharmaceutical Oncology certification of Accredited Pharmacist of Ambulatory Cancer Chemotherapy
5. Other board pharmacy certifications (   )

Q5) Subjective, self-assessed degree of confidence in palliative care ability
(no confidence, 0; full confidence, 10)

Q6) Number of attendances at nationwide continuing education sessions related to palliative care per year
1. Zero **2.** 1 to 3 **3.** 4 to 6 **4.** 7 to 9 **5.** More than 9

Q7) Percentage of cancer patients among all your managed patients
1. Zero **2.** Less than 40% **3.** 40% to 69% **4.** 70% to 99% **5.** 100%

Please answer about cancer patients you managed who used opioids during February and March, 2018

Q7) How many of your cancer patients used opioids during the two-month study period?

Q8) What percentage of patients referred to in Q7 above regularly used 6 or more medications?
1. Zero **2.** 1% to 39% **3.** 40% to 69% **4.** 70% to 99% **5.** 100%

Q9) What percentage of patients referred to in Q8 above received inappropriate prescriptions from their doctors?
(Inappropriate prescription is defined as duplication of allogeneic equivalent drugs, prescription of drugs suspected of having drug interactions, prescriptions that deviated from drug package inserts and guidelines, prescriptions that the pharmacist judged were inappropriate, or prescription of drugs with suspected side effects)
1. Zero **2.** 1% to 39% **3.** 40% to 69% **4.** 70% to 99% **5.** 100%

Please select the reasons these prescriptions were inappropriate (multiple answers allowed)
□ Medication-mediated duplication of pharmacological effect
□ Medication-induced drug-drug interactions
□ Unnecessary medications
□ Adverse drug reactions caused by medications
□ Other (please describe the case)

Q10) What percentage of patients referred to in Q8 above were able to reduce the number of regularly used medications due to pharmacist recommendations?
1. Zero **2.** 1% to 39% **3.** 40% to 69% **4.** 70% to 99% **5.** 100%

Please select the reasons for pharmacist recommendations to reduce medications (multiple answers allowed)
□ Medication-mediated duplication of pharmacological effect
□ Medication-induced drug-drug interactions
□ Unnecessary medications
□ Adverse drug reactions caused by medications
□ Change in drug form due to oral feeding difficulty
□ Other (please describe the case)

Q11) Among patients referred to in Q10 above, what was the average number of medications that were reduced by pharmacist recommendations?

1. 0 **2.** 1 **3.** 2 **4.** 3 **5.** 4 **6.** 5 or more

Describe what type of regularly used drugs were reduced by pharmacist recommendations (multiple answers allowed)

Analgesics (□ Acetaminophen □ Non-steroid anti-inflammatory drug □ Opioid □ Analgesic adjuvant)

Hypnotic sedatives (□ Benzodiazepine □ Non-benzodiazepine □ Other ）

Antipsychotics (□ Typical antipsychotic □ Atypical antipsychotic)

Gastrointestinal medications (□ Gastric antacid □ Proton pump inhibitor □ Histamine H_2 receptor blocker □ Prostaglandin □ Other)

Laxatives (□ Salt-based laxative □ Peroral stimulative laxative □ Small intestine irritation laxative □ Enema □ Other)

Antiemetics (□ Dopamine receptor antagonist □ Prokinetic agent □ Antihistamine agent □ Other)

Other (please provide the name of the medicines)

Q12) Please describe any cases among opioid-using patients in which a patient experienced an improvement in adverse drug reactions due to a pharmacist's intervention.

Please answer with regard to cancer patients under your management who did not use opioids during February and March 2018

Q13) How many cancer patients who used opioids during the two-month study period?

Q14) What percentage of patients referred to in Q13 above regularly used 6 or more medications?

1. Zero **2.** 1% to 39% **3.** 40% to 69% **4.** 70% to 99% **5.** 100%

Q15) What percentage of patients referred to in Q14 above received inappropriate prescriptions from their doctors?

(Inappropriate prescription is defined as duplication of allogeneic equivalent drugs, drugs suspected of having drug interactions, drugs that deviated from the package inserts and guidelines, drugs that were presumptively prescribed or drugs with suspected side effects)

1. Zero **2.** 1% to 39% **3.** 40% to 69% **4.** 70% to 99% **5.** 100%

Please select the reason these prescriptions were inappropriate (multiple answers allowed)

□ Medication-mediated duplication of pharmacological effect
□ Medication-induced drug-drug interactions
□ Unnecessary medications
□ Adverse drug reactions caused by medications
□ Other (please describe the case)

Q16) What percentage of patients referred to in Q14 above were able to reduce their regular use of medications due to pharmacist recommendations?

1. Zero **2.** 1% to 39% **3.** 40% to 69% **4.** 70% to 99% **5.** 100%

Please select the reasons for pharmacist recommendations to reduce medications (multiple answers allowed)

□ Medication-mediated duplication of pharmacological effect
□ Medication-induced drug-drug interactions
□ Unnecessary medications
□ Adverse drug reactions caused by medications
□ Change in drug form due to oral feeding difficulty
□ Other (please describe the case)

Q17) Among patients referred to in Q16 above, what was the average number of medications that were reduced by pharmacist recommendations?

1. 0 **2.** 1 **3.** 2 **4.** 3 **5.** 4 **6.** 5 or more

Describe what type of regularly used drugs were reduced by pharmacist recommendations (multiple answers allowed)

Analgesics (□ Acetaminophen □ Non-steroid anti-inflammatory drug □ Opioid □ Analgesic adjuvant)

Hypnotic sedatives (□ Benzodiazepine □ Non-benzodiazepine □ Other)

Antipsychotics (□ Typical antipsychotic □ Atypical antipsychotic)

Gastrointestinal medications (□ Gastric antacid □ Proton pump inhibitor □ Histamine H_2 receptor blocker □ Prostaglandin □ Other)

Laxatives (□ Salt-based laxative □ Peroral stimulative laxative □ Small intestine irritation laxative □ Enema □ Other)

Antiemetics (□ Dopamine receptor antagonist □ Prokinetic agent □ Antihistamine agent □ Other)

Other (please provide the name of the medicines)

Q18) Please describe any cases among opioid-using patients in which a patient experienced an improvement in adverse drug reactions due to a pharmacist's intervention.
